# Pulse Arrival Time and Pulse Interval as Accurate Markers to Detect Mechanical Alternans

**DOI:** 10.1007/s10439-019-02221-4

**Published:** 2019-02-12

**Authors:** Stefan van Duijvenboden, Ben Hanson, Nick Child, Pier D. Lambiase, Christopher A. Rinaldi, Gill Jaswinder, Peter Taggart, Michele Orini

**Affiliations:** 1 Institute of Cardiovascular Science, University College London, London, UK; 2 Clinical Pharmacology, William Harvey Research Institute, Barts and The London School of Medicine and Dentistry, Queen Mary University of London, London, UK; 3 Department of Mechanical Engineering, University College London, London, UK; 4 Department of Cardiology, Guy’s and St. Thomas’s Hospital, London, UK; 5 Barts Heart Centre, St Bartholomews Hospital, London, UK

**Keywords:** Mechancial alternans, Pulse arrival time, Pulse transit time, Electrical alternans

## Abstract

Mechanical alternans (MA) is a powerful predictor of adverse prognosis in patients with heart failure and cardiomyopathy, but its use remains limited due to the need of invasive continuous arterial pressure recordings. This study aims to assess novel cardiovascular correlates of MA in the intact human heart to facilitate affordable and non-invasive detection of MA and advance our understanding of the underlying pathophysiology. Arterial pressure, respiration, and ECG were recorded in 12 subjects with healthy ventricles during voluntarily controlled breathing at different respiratory rate, before and after administration of beta-blockers. MA was induced by ventricular pacing. A total of 67 recordings lasting approximately 90 s each were analyzed. Mechanical alternans (MA) was measured in the systolic blood pressure. We studied cardiovascular correlates of MA, including maximum pressure rise during systole (dPdt_max_), pulse arrival time (PAT), pulse wave interval (PI), RR interval (RRI), ECG QRS complexes and T-waves. MA was detected in 30% of the analyzed recordings. Beta-blockade significantly reduced MA prevalence (from 50 to 11%, *p* < 0.05). Binary classification showed that MA was detected by alternans in *dPdt*_max_ (100% sens, 96% spec), PAT (100% sens, 81% spec) and PI (80% sens, 81% spec). Alternans in PAT and in PI also showed high degree of temporal synchronization with MA (80 ± 33 and 73 ± 40%, respectively). These data suggest that cardiac contractility is a primary factor in the establishment of MA. Our findings show that MA was highly correlated with invasive measurements of PAT and PI. Since PAT and PI can be estimated using non-invasive technologies, these markers could potentially enable affordable MA detection for risk-prediction.

**Electronic supplementary material:**

The online version of this article (10.1007/s10439-019-02221-4) contains supplementary material, which is available to authorized users.

## Introduction

Mechanical alternans (MA), also known as pulsus alternans, is defined as a blood pressure change occurring on an every other beat basis, which can manifest in a succession of strong and weak pulses. Since its discovery in the late nineteenth century,^17^ it has been recognized as a marker of cardiovascular impairment given the association with increased mortality in heart failure^11^ and idiopathic dilated cardiomyopathy.^9^,^14^

Mechanical alternans is a manifestation of broader phenomenon known as cardiac alternans, which involves the excitation–contraction coupling and includes electrical alternans, a phenomenon describing electrical conduction and/or repolarization changes on an every other beat basis. Electrical alternans manifests as a beat to beat change in the T-wave and/or QRS complex morphology, known as T-wave alternans (TWA) and/or QRS alternans (QRSA), respectively, which are established predictors of sudden cardiac death.^4^,^17^,^26^,^31^,^33^

The interaction between mechanical and electrical alternans is still unclear, mainly due to the limited number of studies that have included simultaneous recordings of both types of alternans. Previous work has reported both strong^27^ and moderate^10^,^11^,^13^ correlations between mechanical and electrical alternans. Mechanical and electrical alternans may be driven by different mechanisms, with the former due to hemodynamic and/or inotropic alterations^29^ and the latter due to discrete local disturbances within the conduction system or myocardium^17^,^33^ providing complementary information about cardiac vulnerability.

The use of MA as a risk stratification marker is appealing but limited by the invasiveness of arterial pressure recordings and the cost of non-invasive continuous blood pressure monitors. The aim of this study is to assess novel cardiovascular correlates of MA in the intact human heart using invasive derived measurements to advance our understanding of its pathophysiology to facilitate affordable non-invasive detection.

## Methods

Continuous and synchronous recordings of invasive arterial blood pressure, respiration (chest movement) and the limb-lead electrograms (ECGs) were obtained from 12 subjects before routine clinical radiofrequency ablation procedures for atrial fibrillation in the cardiac catheterization lab at the St Thomas’ Hospital in London, as described previously.^8^,^30^

All subjects had normal ventricular function and none of them were known to have ventricular scar or disordered conduction due to bundle branch abnormalities. Studies were performed in the unsedated state and cardio-active medications were discontinued for 5 days before the study.

Arterial blood pressure was measured from a femoral artery with a continuous-flush pressure transducer system (Tru-Wave PX600F; Ed-wards Lifesciences, Irvine, CA). The subject’s breathing cycle was monitored using a custom-constructed tension sensor fixed to a freely expandable band placed around the chest/abdomen (adapted from a RESPeRATE device; InterCure, New York, NY). ECG, blood pressure and respiration were synchronously recorded and digitized at 1200 Hz (Ensite 3000; Endocardial Solutions).

### Experimental Protocol

During the study, heart rate was clamped by right ventricular pacing using a Biotronik (Berlin, Germany) stimulator (model UHS 3000) throughout the experiment. Mechanical alternans was induced by ventricular pacing, which was applied at twice the diastolic threshold and 2 ms pulse width, at a cycle length > 20 beats/min faster than the intrinsic AF rate (median of pacing interval equal to 500 ms). Breathing was regulated voluntary at four fixed rates (6, 9, 15, and 30 breaths/min) for 90 s each, in random order. The subjects were instructed by a video monitor that displayed a computer-generated animation of lung volume (implemented in LabVIEW software; National Instruments, Austin, TX). Paced breathing was practiced before study commencement. The breathing protocol was first performed under control conditions. All patients then received beta-blocker (metoprolol) intravenously and the breathing protocol was repeated. This resulted in a total of 12 × 4 × 2 = 96 recording of 90 s duration with controlled breathing and heart rate.

### Analysis of Recordings

**Figure 1:**
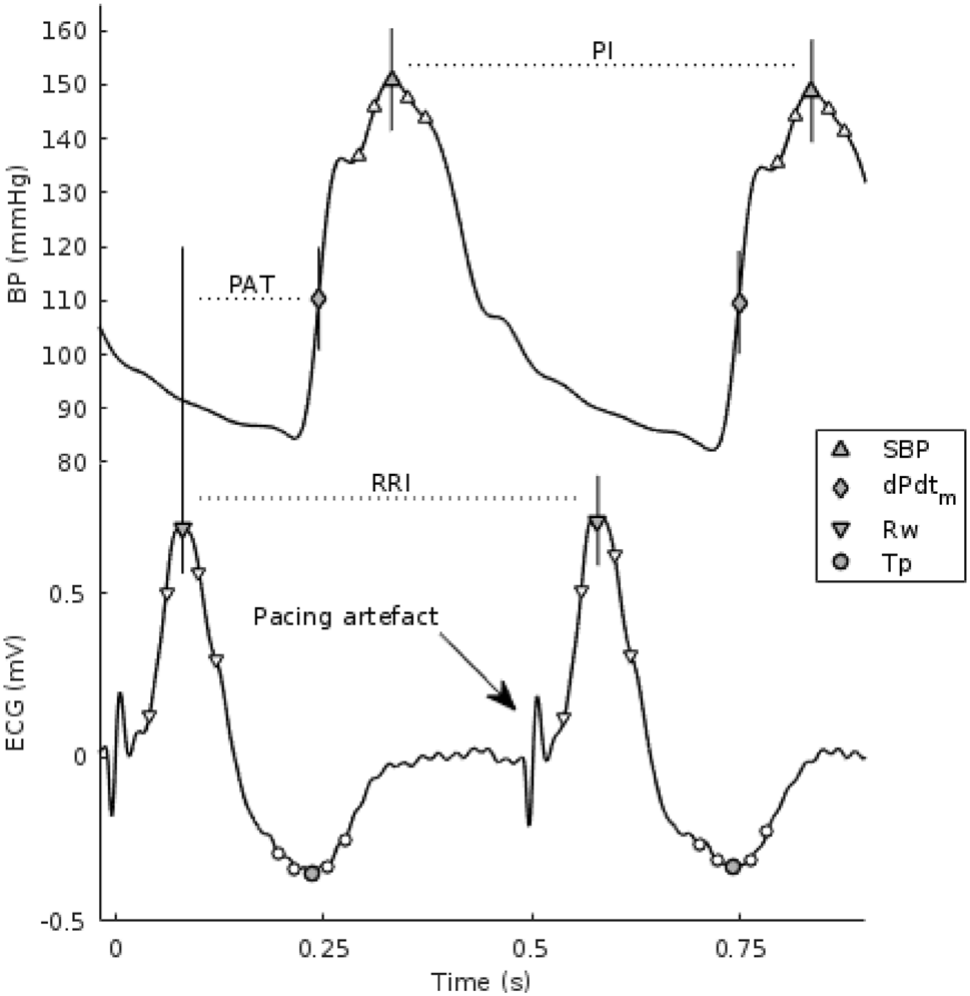
Overview of the obtained measurements from the blood pressure and ECG waveforms. *SBP* systolic blood pressure, *dPdt*_*m*_ (*dP*/*dt*)max, *Rw* peak of the R-wave, *Tp* peak of the T-wave, *PI* pulse interval, *PAT* pulse arrival time, *RRI* RR interval. Note the broad QRS complexes as a result of ventricular pacing (corresponding pacing artefacts are visible at *t* = 0 s and *t* = 0.5 s).

Measurements were analyzed offline. ECG and arterial pressure signals were band-pass filtered between 0.5 and 35 Hz to attenuate noise. The signals were then used to determine the following indices (Fig. 1):

1. Systolic blood pressure (SBP) as well as blood pressure measured 8 and 16 ms before and after the systolic peak (total of 5 blood pressure measures per pulse).
2. The maximum upstroke of the pulse (*dPdt*_max_), computed as the maximum derivative of the blood pressure series during systole.
3. T-wave amplitude at the peak of the T-wave as well as T-wave amplitude measured 8 and 16 ms before and after the T-wave peak (total of 5 measures per T-wave).
4. QRS-wave amplitude as well as QRS-wave amplitude measured 8 and 16 ms before and after the QRS peak (total of 5 measures per QRS complex). The QRS wave amplitude was measured as the R-wave amplitude for positive QRS complexes, or S wave amplitude for negative QRS complexes.
5. RR interval, as the interval between two consecutive R-waves in the ECG lead measured from the best signal quality among leads I, II and III.
6. Pulse wave interval (PI), as the interval between systolic peaks of consecutive beats.
7. Pulse arrival time (PAT), measured as the interval between the occurrence of the R-wave peak and the *dPdt*_max_ within the same heartbeat.

Beats for which any of these measurements could not be determined (e.g. ectopic beats) were replaced by the mean value of the series. Series were rejected if the interpolated values constituted more than 10% of the total number of measurements.

## Alternans Detection

Alternans was detected using a standard spectral method.^19^,^24^ First, a detrending filter was used to enhance beat-to-beat changes:

$$Y_{D} \left[ {n,b} \right] = Y\left[ {n,b} \right] - Y[n,b - 1]$$

where *Y* represents the time series of one of the investigated markers (SBP, dPdt_max_, QRS, T-wave amplitude, RRI, PI or PAT), with *n* = 1:N being the number of samples taken from each marker within each beat, and *b* = 1:B the number of beats. As shown in Fig. 1, *N* = 5 for SBP, QRS-wave and T-wave amplitudes, while *N* = 1 for RRI, dPdt_max_, PI and PAT. The Fourier transform was used to estimate the power-spectrum of $Y_{D} \left[ {n,b} \right]$(along *b*) using a moving window L beats wide and centered at heartbeat $b$:

$$P_{alt}^{Y} \left( {b,f} \right) = \frac{{\mathop \sum \nolimits_{n}^{N} \frac{1}{{L^{2} }} \left| {\mathop \sum \nolimits_{{l = - \frac{L}{2} + 1}}^{L/2} Y_{D} [b + l,n]e^{ - 2\pi il} } \right|^{2} }}{N}$$

The alternans magnitude $M_{alt}^{Y} (b)$ at beat $b$ was then computed by taking the square root of the sum of $P_{alt}^{Y} \left( {b,f} \right)$ between 0.46 and 0.5 cycles/beat^1^,^28^:

$$M_{alt}^{Y} \left( b \right) = \sqrt {\mathop \sum \limits_{{\begin{array}{*{20}c} {0.46 < f \le 0.5} \\ \\ \end{array} }} P_{alt}^{Y} \left( {b,f} \right)}$$

Results will be given for *L* = 16, 32 and 64 beats.

### Statistical Analysis

The interaction between MA and other markers was assessed both in terms of inter-recordings binary classification (whether a recording can be correctly classified as showing or not-showing mechanical alternans) and intra-recording temporal correlation (the degree of synchronization between mechanical alternans and alternans in the investigated markers).

The inter-recordings binary classification was used to investigate the accuracy of each of the investigated markers to detect recordings with and without MA. The sensitivity of each marker to predict MA was defined as the fraction of the recordings with MA that were correctly identified, whereas the specificity was defined as the fraction of recordings without MA that were correctly identified. A recording was classified as MA positive if the beat-to-beat SBP magnitude, $M_{alt}^{SBP} (b)$, exceeded the alternans threshold of 4 mmHg, i.e. $M_{alt}^{SBP} \left( b \right) > 4\,{\text{mmHg}}$, for at least 5% of the total number of beats within the whole recording. Alternans in the investigated markers, $Y_{D}$, was detected when $M_{alt}^{Y} (b)$ exceeded a threshold for at least 5% of the total number of beats within the whole recording. The optimal threshold was determined using ROC analysis. Recordings were divided into a training and a testing data set, each one containing the same number of recordings from different patients to ensure independence.

For each cardiovascular correlate, the optimal threshold to detect MA was estimated in the training set as the threshold that corresponded to the point of the ROC curve that maximised the product sensitivity × specificity.

The intra-recording temporal correlation between the investigated markers and MA was quantified by computing temporal sensitivity (*t*Sens) and specificity (*t*Spec). Temporal sensitivity, $tSens^{XY}$, was defined as the duration of the interval during which alternans was simultaneously present in $X(b)$ and $Y(b)$ divided by the duration of the interval during which alternans was present in $Y(b)$. Temporal specificity, $tSpec^{XY}$, was defined as the duration of the interval during which alternans was simultaneously absent in $X(b)$ and $Y(b)$ divided by the duration of the interval during which alternans was absent in $Y(b)$.

**Figure 2:**
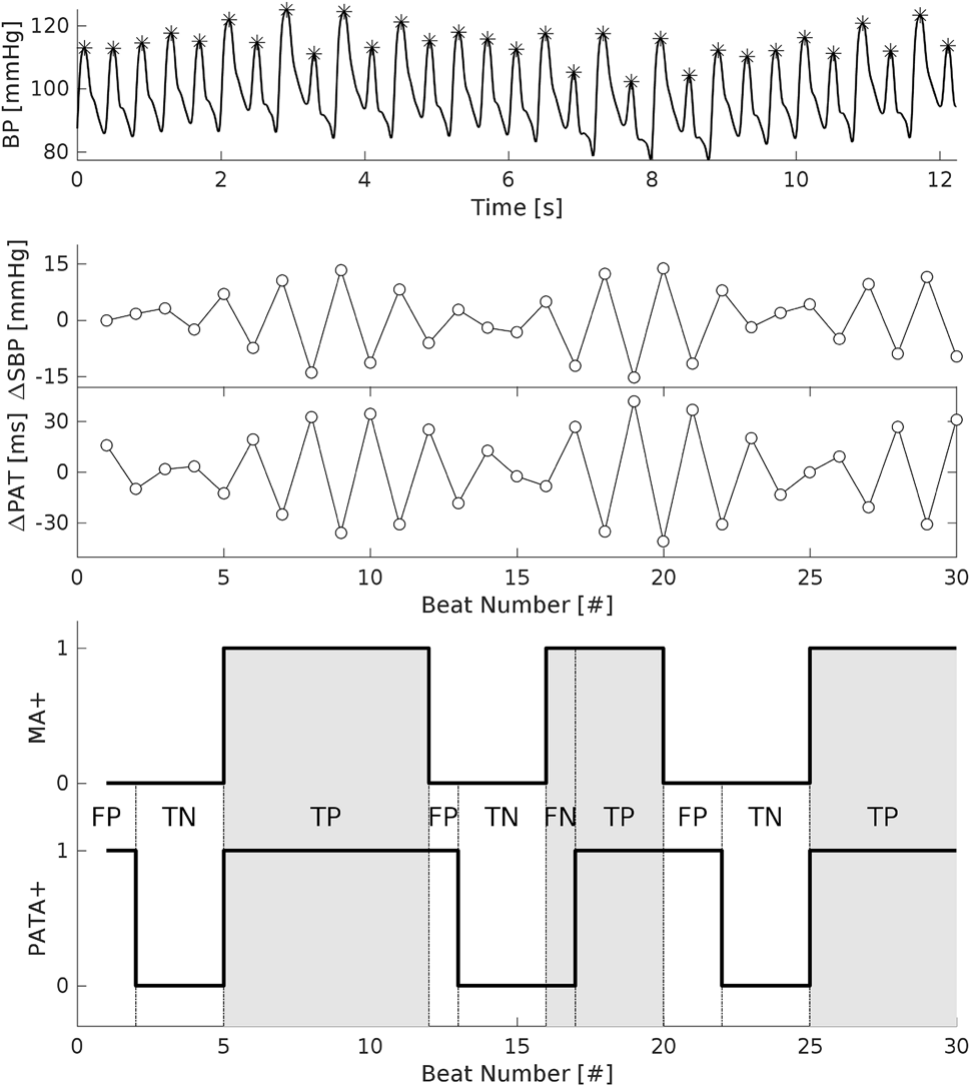
Temporal agreement between mechanical alternans (MA) and alternans in the pulse arrival time (PATA). Upper panel shows the raw BP trace with temporal episodes of MA around 10 and 20 s. The lower panel show the temporal agreement between MA (indicated by grey shading) and PATA: *TP* true positive: alternans in both systolic blood pressure (SBP) and PAT, *TN* true negative: absence of alternans in both markers, *FP* false positive: PATA during absence of MA, *FN* false negative: Absence of PATA during MA.

Figure 2 shows an example of the temporal agreement between MA and PAT alternans (PATA) with $tSens^{PATA,MA} = 0.95$ (95% temporal overlap between PATA and MA) and $tSpec^{PATA,MA} = 0.74$ (74% of temporal overlap between absence of PATA and absence of MA, indicating the presence of PATA at times when MA was absent).

The Fisher exact probability test was used to test whether the proportion of recordings with MA was statistically different across different respiratory rates. We applied McNemar *χ*^2^ test for paired nominal data to compare the proportion of recordings with MA during control and following administration of beta-blocker.

## Results

From the 96 breathing episodes, pressure recordings were available in 84, from which 67 recordings (80%) were eligible for analysis while 17 recordings were discarded due to high number of ectopic beats (number of ectopics exceeding 10% of total number of beats). Administration of beta-blocker was associated with a significant reduction of the mean dPdt_max_: 998 ± 383 vs. 797 ± 331 mmHg/s, *p* = 0.004, whereas SBP showed a tendency to decrease: 138 ± 19 vs. 130 ± 25 mmHg, *p* = 0.7.

**Figure 3:**
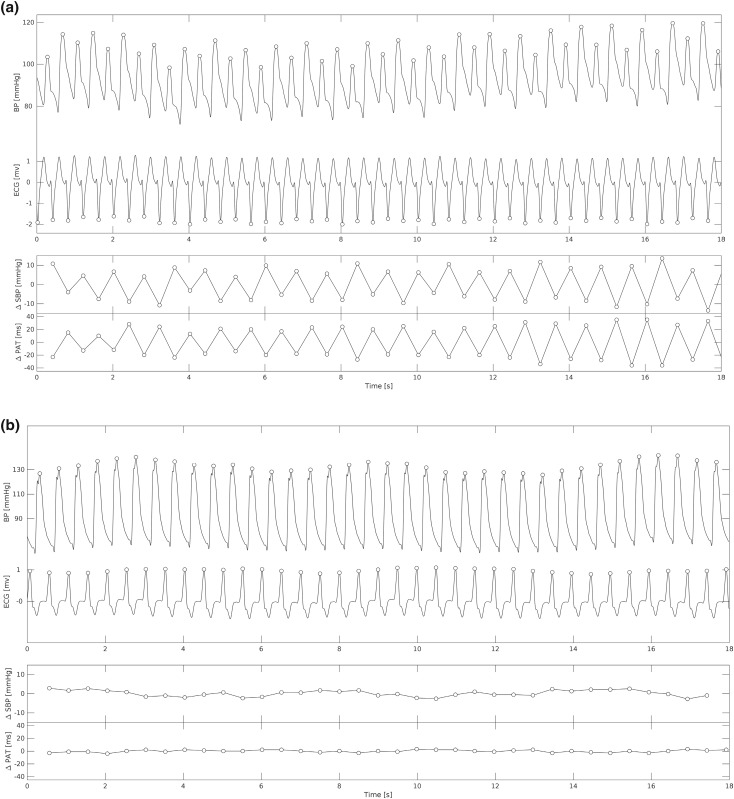
Examples of blood pressure and ECG traces during presence of MA (**a**) and absence (**b**). *SBP* systolic blood pressure, *PAT* pulse arrival time.

**Figure 4:**
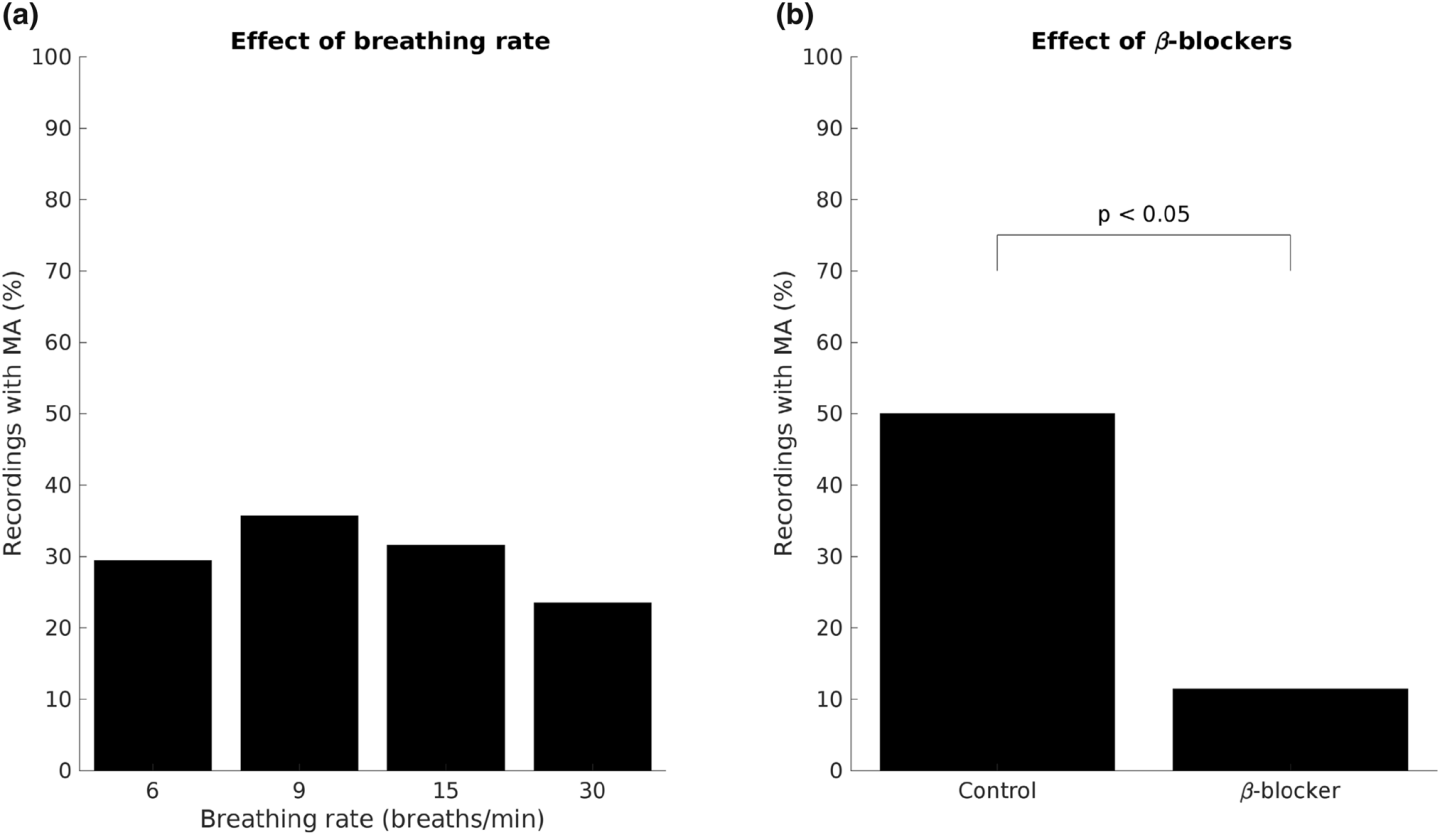
Prevalence of MA for respiratory frequency (**a**) and control vs. beta-blockade (**b**).

Examples of recordings with and without MA are shown in Fig. 3. Episodes of MA were observed in 7 out of 12 subjects and detected in 30% of the available recordings. No significant differences were found in the proportions of recordings with MA across different respiratory rates (*p* = 0.8, Fig. 4a). However, administration of beta-blocker was associated with a significantly reduced the proportion of recordings with MA from 50 to 11% (p < 0.05) (Fig. 4b).

**Figure 5:**
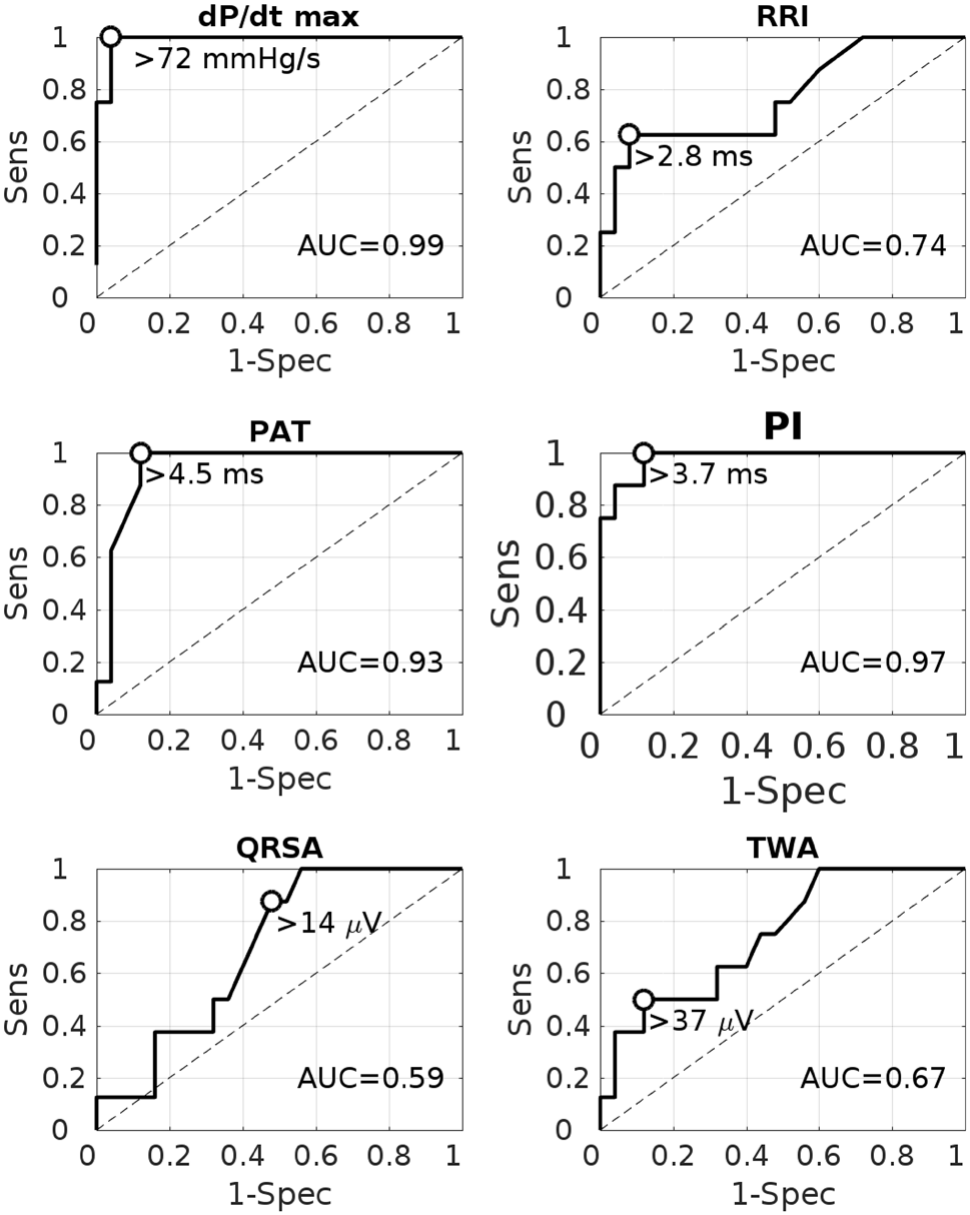
ROC curves for tested markers to predict recordings with mechanical alternans. *RRI* RR interval, *PAT* pulse arrival time, *PI* pulse wave interval, *QRSA* QRS-complex alternans, *TWA* T-wave alternans.

The results of alternans inter-recordings binary classification analysis are shown in Fig. 5 and Table 1. Alternans was present in all markers with similar prevalence (Table 1), which was however highest for PATA and PIA: 43 and 37%, respectively. Alternans in dPdt_max_ showed the strongest correlation with MA with an accuracy of 97% (100% sensitivity, 96% specificity). The corresponding optimal alternans threshold to predict MA was 72 mmHg/s. PAT alternans (PATA) and PI alternans (PIA) detected MA with accuracies equal to 87% (100% sensitivity, 81% specificity) and 81% (80% sensitivity, 81% specificity), respectively, and optimal thresholds equal to 4.5 and 3.7 ms, respectively. The linear correlation between PATA and MA magnitudes was modest but significant (*ρ* = 0.63, *p* < 0.001). No significant correlation was found between MA and PIA magnitudes (*ρ* = 0.29, *p* = 0.13). Moderate to high inter-recordings classification accuracy were observed for RRI alternans (RRIA, acc = 78%), TWA (acc = 79%) and QRSA (acc = 70%), see Table 1. The corresponding alternans thresholds for optimal accuracy to detect MA were 2.8 ms, 37, and 14 *µ*V, respectively. It is worth noting that very similar results were obtained when using the full data-set for both training and testing (Supplementary Fig. 1 and Supplementary Table 1).

**Table 1:** Accuracy, sensitivity, and specificity of investigated cardiovascular markers to detect mechanical alternans evaluated in the testing data-set.

	Prev. (%)	MA	DPDTA	PATA	PIA	RRIA	TWA	QRSA
MA	30	1 (1/1)	0.97 (1/0.96)	0.87 (1/0.81)	0.81 (0.8/0.81)	0.78 (0.5/0.89)	0.79 (0.85/0.77)	0.7 (0.3/0.87)
DPDTA	33		1 (1/1)	0.87 (0.95/0.82)	0.84 (0.82/0.84)	0.75 (0.45/0.89)	0.79 (0.82/0.78)	0.67 (0.27/0.87)
PATA	43			1 (1/1)	0.76 (0.66/0.84)	0.64 (0.34/0.87)	0.81 (0.76/0.84)	0.63 (0.28/0.89)
PIA	37				1 (1/1)	0.73 (0.44/0.9)	0.72 (0.68/0.74)	0.66 (0.28/0.88)
RRIA	22					1 (1/1)	0.69 (0.73/0.67)	0.78 (0.4/0.88)
TWA	42						1 (1/1)	0.73 (0.39/0.97)
QRSA	18							1 (1/1)

*MA* mechanical alternans, *DPDTA* dPdt_max_ alternans, *PATA* pulse arrival time alternans, *PIA* pulse wave interval alternans, *TWA* T-wave alternans, *QRSA* QRS-complex alternans

**Figure 6:**
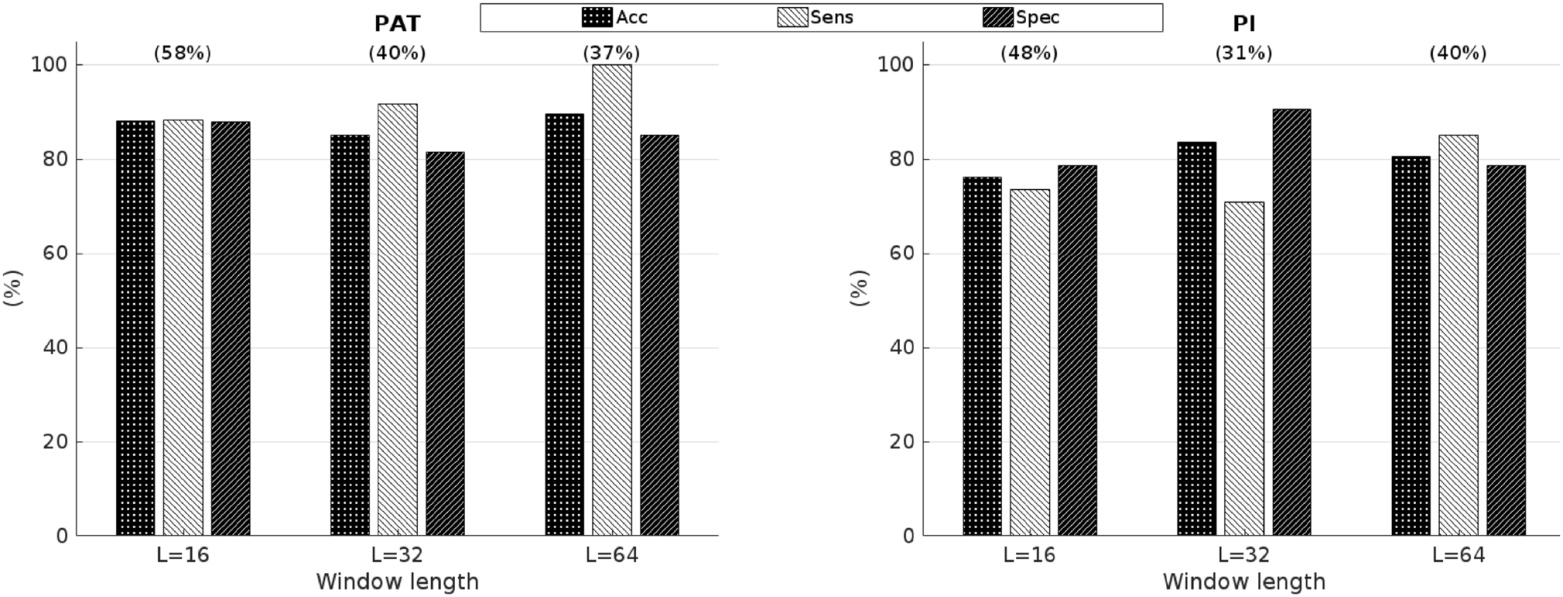
Accuracy (Acc), sensitivity (Sens), and specificity (Spec) to predict mechanical alternans using alternans in pulse arrival time (PAT) and pulse width interval (PIA) for different analysis window lengths (L). The percentage of recordings with alternans is shown between brackets on top of the bars.

The effect of different window lengths of the sliding window for alternans detection on the accuracy of PATA and PIA is shown in Fig. 6. No major differences were found. However, the sensitivity of PATA to detect MA decreased from 92% for *L* = 32 beats to 88% for *L* = 16 beats.

The temporal intra-recording correlation between markers of alternans is presented in Table 2. There was a moderate to high temporal synchronization with MA for PATA $\left( {tSens^{PATA,MA} \, = \, 80\, \pm \, 3 3\% } \right)$ and PIA $\left( {tSens^{PIA,MA} \, = \, 7 3\, \pm \, 40\% } \right)$, whereas mean $tSens^{TWA,MA}$, $tSens^{QRSA,MA}$, and $tSens^{RRIA,MA}$ were within the range 48–64%.

**Table 2:** Temporal agreement between mechanical alternans and alternans of the investigated cardiovascular markers: mean tSens ± standard deviation and mean tSpec ± standard deviation.

	tSens	tSpec
DPDTA	0.93(± 0.15)	0.7(± 0.38)
PATA	0.8(± 0.3)	0.74(± 0.38)
PIA	0.73(± 0.4)	0.54(± 0.43)
RRIA	0.48(± 0.46)	0.68(± 0.46)
QRSA	0.51(± 0.43)	0.62(± 0.43)
TWA	0.64(± 0.42)	0.55(± 0.43)

For abbreviations see Table 1

## Discussion

The aim of this study was to advance our understanding of the pathophysiology of MA and facilitate affordable non-invasive detection by studying its correlation with other cardiovascular markers in the intact human heart. The main findings are: (1) Pacing-induced MA is almost always accompanied by alternans in dPdt_max_ and the prevalence decreased following beta-blocker administration; (2) Alternans in PAT and PI were highly correlated with MA (accuracy > 0.84); (3) Moderate correlation was found between MA and RRIA, TWA and QRSA.

The first main finding indicates that cardiac contractility is a primary factor in the establishment of MA. It was observed that the cardiovascular markers with better correlation with MA are all associated with contractility: dPdt_max_ is considered a marker of cardiac contractility in itself, while PAT and PI strongly depend on the pre-ejection period and pulse wave velocity, which in turn depend on contractility. Furthermore, we observed a significant decrease in MA prevalence after beta-adrenergic blockade. Since beta-blockers reduce cardiac inotropy, this further supports a direct link between MA and cardiac contractility and is in line with previous work showing a reduction in MA occurrence in patients with chronic heart failure following long-term beta-blocker therapy.^12^

Regarding the second main finding, the similar accuracy with which PAT and PI detected MA is most probably due to the fact that PAT is an important component of PI.^32^ The analysis of intra-recording temporal correlation showed that PAT and PI were not only able to correctly classify recordings as MA positive or negative but they also offer the possibility of tracking MA intermittent behavior with good accuracy. The PAT is equal to the sum of PTT, the time interval required for a pressure wave to travel between two sites (typically between a proximal site and a distal site) in the arterial tree, and the pre-ejection period. The pre-ejection period is determined by the ventricular electro-mechanical delay and isovolumic contraction period.^6^ Therefore, the close association between MA and PAT alternans is likely to be mediated by beat-to-beat fluctuations in the pre-ejection period induced by changes in contractility. The possibility of measuring blood pressure non-invasively through either the PTT or the PAT has been extensively investigated, but it remains controversial.^32^ The results of this study indicate that although PAT may not provide accurate estimates of SBP (at least without calibration), it can accurately track rapid oscillations in SBP. An important implication is that MA can be detected by analyzing beat to beat variations in the pulse interval, which can be measured non-invasively with affordable technology such as for example photoplethysmography, providing a potential surrogate for MA as an independent predictor of cardiovascular mortality without the need of invasive recordings and expensive technology.^3^

The moderate correlation (75%) between MA and RRIA was unexpected since the studies were conducted during ventricular pacing at a fixed heart rate. This may be due to an interaction between the pre-ejection period and RRI, mediated by conduction velocity changes which reflect in changes in the QRS morphology. Another possible link between MA and alternans in the RRI may be mediated by the baroreflex.^15^,^22^ The weakest correlation with MA was found for QRS and T-wave alternans, which is in line with previous observations that T-wave alternans can occur in the absence of MA and *vice versa*.^10^,^13^

This study has a balanced number of recordings with and without MA, which is important for accurate sensitivity and specificity estimation. Accuracy was measured in a testing data-set independent of the training data-set. Similar results were obtained when using the full data-set for both training and testing. A likely explanation for the relatively high prevalence of MA may be the relatively high pacing rate (median cycle length was equal to 500 ms). One of the proposed mechanisms underlying the establishment of MA is based on the hypothesis that a sequence of weak and strong beats is maintained by the succession of these events: a beat associated with low end-diastolic volume contracts with low force due to Frank-Starling mechanism and has high end-systolic volume (weak beat). The following beat has high end-diastolic volume and contracts with high force (strong beat) and therefore has low end-systolic volume. The next beat will have low-end diastolic volume and low force and so on. The short cycle length as a result of the high pacing rate would accentuate this process because it reduces the time available for diastolic filling.^5^ Previous studies have indeed shown that MA is more prevalent and likely to be sustained at higher heart rates.^5^,^9^ Furthermore, ventricular pacing alters contractility which may also facilitate MA induction. The prevalence of RRIA and QRSA was lower in the training than in the full data-set. This is probably due to the weak association between these markers and MA, which makes the estimation of the best threshold for MA detection through ROC analysis unreliable.

The main focus of this work was to explore the possibility of indirectly detect MA using pulse arrival time and pulse interval. Further studies are needed to determine the best estimator for mechanical alternans. In this study, we have implemented a modified spectral method in which MA is detected by comparing the spectral power in an *ad-hoc *alternans spectral band with the absolute threshold (4 mmHg) consistently used in previous clinical work.^9^,^11^–^13^ The alternans’ spectral band was extended to include frequencies within 0.46–0.5 Hz to reduce the effect of phase resetting, where a change in the alternans phase results in a shift of the alternans spectral peak toward slightly lower frequencies.^1^ Future research should focus on the optimization of mechanical alternans detection by testing different advanced methodologies proposed for T-wave alternans detection^16^,^19^–^21^ including how to compensate for phase resetting in the alternans sequence due to ventricular premature contractions.^20^

Previous studies have demonstrated that the photoplethysmographic signal can be used to measure other important markers of cardiovascular risk such as heart rate variability^7^ respiratory rate,^23^ heart rate turbulence,^2^ atrial fibrillation^18^*etc*. The integration of markers related to different mechanisms of cardiac instability has been shown to improve cardiac risk prediction,^25^ and the combination of MA with other markers of risk derived from PI analysis should be investigated in future studies.

## Conclusion

The results from this study indicate cardiac contractility as a primary factor in the establishment of MA. We demonstrated that invasive derived measurements of PATA and PIA are able to detect MA with high accuracy. Future studies should investigate whether PAT and PI measured by non-invasive and affordable technology such as photoplethysmography, currently widely incorporated in wearable devices, can also provide accurate estimation of MA.

## Electronic supplementary material

Below is the link to the electronic supplementary material.

Supplementary material 1 (PDF 221 kb)
