# Supplementary material for: Pulse Arrival Time and Pulse Interval as Accurate Markers to Detect Mechanical Alternans
Source: Ann Biomed Eng. 2019 Feb 12;47(5):1291–9. doi: 10.1007/s10439-019-02221-4 (PMC6453876; doi:10.1007/s10439-019-02221-4)
Supplement: Supplementary file 1 — Supplementary material 1 (PDF 221 kb) [file 10439_2019_2221_MOESM1_ESM.pdf]

Pulse Arrival Time and Pulse Interval as Accurate Markers to Detect Mechanical Alternans

Supplementary data

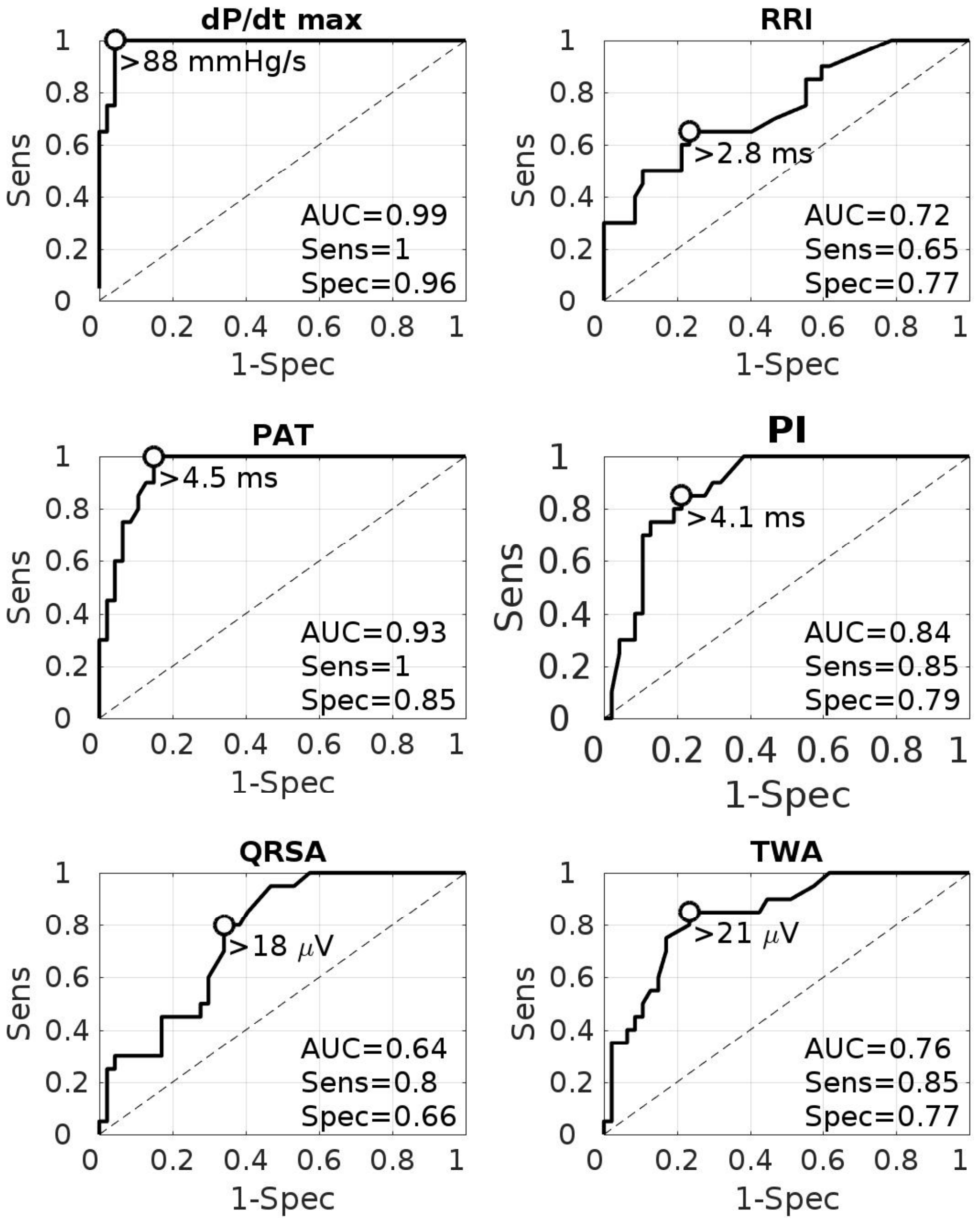

Supplementary Figure 1: *ROC curves to determine the optimal thresholds for MA detection. Sensitivity and specificity were evaluated in the full data-set. The white bullet on the ROC curve indicates the point for which the threshold to predict MA was optimal. RRI: RR interval, PAT: Pulse Arrival Time, PI: Pulse Wave Interval, QRSA: QRS-complex alternans, TWA: T-wave alternans.*

Row	Prev. (%)	MA	DPDTA	PATA	PIA	RRIA	TWA	QRSA
MA	30	1 (1/1)	0.97 (1/0.96)	0.9 (1/0.85)	0.81 (0.85/0.79)	0.73 (0.65/0.77)	0.79 (0.85/0.77)	0.7 (0.8/0.66)
DPDTA	33		1 (1/1)	0.87 (0.91/0.84)	0.84 (0.86/0.82)	0.73 (0.64/0.78)	0.79 (0.82/0.78)	0.67 (0.73/0.64)
PATA	40			1 (1/1)	0.76 (0.7/0.8)	0.66 (0.52/0.75)	0.81 (0.78/0.82)	0.78 (0.81/0.75)
PIA	40				1 (1/1)	0.75 (0.63/0.82)	0.69 (0.63/0.72)	0.6 (0.59/0.6)
RRIA	36					1 (1/1)	0.64 (0.58/0.67)	0.55 (0.54/0.56)
TWA	42						1 (1/1)	0.88 (0.93/0.85)
QRSA	48							1 (1/1)

Supplementary Table 1: Accuracy, sensitivity, and specificity of investigated cardiovascular markers to detect mechanical alternans evaluated in the full data-set.. MA: Mechanical alternans, DPDTA = $dPdt_{max}$ alternans, PATA: Pulse Arrival Time alternans, PIA: Pulse Wave Interval alternans, TWA: T-wave alternans, QRSA: QRS-complex alternans
